# T cell memory to evolutionarily conserved and shared hemagglutinin epitopes of H1N1 viruses: a pilot scale study

**DOI:** 10.1186/1471-2334-13-204

**Published:** 2013-05-04

**Authors:** Venkata R Duvvuri, Bhargavi Duvvuri, Veronica Jamnik, Jonathan B Gubbay, Jianhong Wu, Gillian E Wu

**Affiliations:** 1 Biology Department, York University, Toronto, Canada; 2 Kinesiology & Health Science, York University, Toronto, Canada; 3 Centre for Disease Modeling, York Institute of Health Research, Toronto, Canada; 4 Ontario Agency for Health Protection and Promotion, Toronto, Canada; 5 Mount Sinai Hospital, Toronto, Canada; 6 University of Toronto, Toronto, Canada; 7 The Hospital for Sick Children, Toronto, Canada

**Keywords:** Influenza A/H1N1 viruses, Hemagglutinin, CD4+ T cell epitope, Immunodominant epitope, Novel conserved HA epitope

## Abstract

**Background:**

The 2009 pandemic influenza was milder than expected. Based on the apparent lack of pre-existing cross-protective antibodies to the A (H1N1)pdm09 strain, it was hypothesized that pre-existing CD4+ T cellular immunity provided the crucial immunity that led to an attenuation of disease severity. We carried out a pilot scale study by conducting *in silico* and *in vitro* T cellular assays in healthy population, to evaluate the pre-existing immunity to A (H1N1)pdm09 strain.

**Methods:**

Large-scale epitope prediction analysis was done by examining the NCBI available (H1N1) HA proteins. NetMHCIIpan, an eptiope prediction tool was used to identify the putative and shared CD4+ T cell epitopes between seasonal H1N1 and A (H1N1)pdm09 strains. To identify the immunogenicity of these putative epitopes, human IFN-γ-ELISPOT assays were conducted using the peripheral blood mononuclear cells from fourteen healthy human donors. All donors were screened for the HLA-DRB1 alleles.

**Results:**

Epitope-specific CD4+ T cellular memory responses (IFN-γ) were generated to highly conserved HA epitopes from majority of the donors (93%). Higher magnitude of the CD4+ T cell responses was observed in the older adults. The study identified two HA2 immunodominant CD4+ T cell epitopes, of which one was found to be novel.

**Conclusions:**

The current study provides a compelling evidence of HA epitope specific CD4+ T cellular memory towards A (H1N1)pdm09 strain. These well-characterized epitopes could recruit alternative immunological pathways to overcome the challenge of annual seasonal flu vaccine escape.

## Background

A pandemic influenza A H1N1 virus, A (H1N1)pdm09, emerged in April 2009 with a unique combination of genes that have not been reported previously in swine or humans [1]. A large proportion of the human population displayed a lack of cross-reactive neutralizing antibodies to A (H1N1)pdm09 virus, a situation accepted to be due to the novel antigenicity of the virus [2]. This immunologically naïve context of the human population was responsible for the rapid global spread of the virus leading the World Health Organization to declare a pandemic on June 11, 2009. Despite being highly pathogenic in laboratory animals, A (H1N1)pdm09 virus unexpectedly turned out to be a milder strain in humans, and was also found to be self-limiting in disease spread in the majority of documented human cases [3,4]. A possible explanation for this unanticipated scenario is the presence of pre-existing T-cellular responses towards the highly conserved protein regions shared by seasonal influenza A H1N1 and A (H1N1)pdm09 that would result in limited virus spread, although not limited establishment of infection [5,6]. The importance of CD4+ T cell responses in influenza infection is well documented. CD4+ T cell responses provide cross-reactive immunity to different influenza strains even in the absence of significant antibody titers; they have a crucial role in maintaining the effector CD8+ T cell functions during a primary response and they are involved in compensating for CD8+ T cells diminished in cytolytic activity, particularly in older adults [7,8]. Given the importance of CD4+ T helper cells in shaping cross-reactive B-cell and T-cell immune responses and the conservation and function of the hemagglutinin (HA) protein in the influenza viral life cycle, we investigated the immunogenic potential of conserved HA CD4+ T cell epitopes, shared between seasonal H1N1 and A (H1N1)pdm09. Our findings lead to the conclusion that recall responses from CD4+ memory T cells generated by previous exposure to seasonal H1N1 viruses and also identifies a highly conserved, novel immunodominant CD4+ T cell epitope that would be a promising target for universal epitope-based influenza vaccines.

## Methods

### Hemagglutinin (HA) protein sequences

Accessing the National Center for Biotechnology Information (NCBI) yielded 924 and 890 HA protein sequences of seasonal H1N1 (1977–2009), and A (H1N1)pdm09 (2009–2010) respectively. BioEdit software program was used for sequence analysis.

### Epitope prediction, selection of optimal epitopes and epitope synthesis

The NetMHCIIpan epitope prediction program was utilized to identify the optimal CD4+ T cell epitopes (length 15 a.a.) from the HA of A(H1N1)pdm09 based on the binding affinities of epitope-MHC II allele complex [9]. The following criteria were applied to select the predicted epitopes from the A (H1N1)pdm09 strain (Table 1): greater than 90% of predicted epitope conservancy in seasonal H1N1(1977–2009) HA proteins, greater than >90% seasonal H1N1(1977–2009) isolates with each of the conserved epitope and greater than >90% A(H1N1)pdm09 (2009–2010) isolates with each of the conserved epitope [10]. Human leukocyte antigen (HLA)-DRB1 alleles were selected because of their wide coverage in the human population (~99%) [11,12]. A total of 18 CD4+ T cell epitopes of HA met the epitope selection criteria, of which 13 epitopes were selected for the experimental analysis randomly (bolded in Table 2). These 13 conserved epitopes were synthesized by Proimmune Inc (Florida, USA), at the recommended purity for ELISPOT assays.

**Table 1:** Predicted CD4+ HA T cell epitopes of A(H1N1)pdm09 viruses and their conservancy in seasonal H1N1 (1977–2009) viruses

**Predicted CD4+ T- cell epitopes (15 a. a) (epitope position)**	**% of predicted epitope conservancy in seasonal H1N1(1977–2009) HA proteins**	**% seasonal H1N1(1977–2009) isolates with each of the conserved epitope**	**% A(H1N1)pdm09 (2009–2010) isolates with each of the conserved epitope**
**VLEKNVTVTHSVNLL**	100	100	99
(HA1 19–33)			
**EELREQLSSVSSFER**	100	100	99
(HA1 99–113)			
**QLSSVSSFERFEIFP**	100	100	99
(104–119)			
**ATGLRNIPSIQSRGL**	93	97	98
(HA1 315–329)			
GLRNIPSIQSRGLFG	100	97	100
(HA1 317–331)			
**RNIPSIQSRGLFGAI**	100	97	98
(HA1 319–333)			
QSRGLFGAIAGFIEG	100	100	100
**(**HA1 325**–**339)			
**NKVNSVIEKMNTQFT**	100	96	99
(HA2 377–391)			
VIEKMNTQFTAVGKE	100	96	99
(HA2 382–396)			
**DGFLDIWTYNAELLV**	100	96	99
(HA2 413–427)			
WTYNAELLVLLENER	100	99	100
(HA2 419–433)			
TYNAELLVLLENERT	100	99	100
(HA2 420–434)			
**YNAELLVLLENERTL**	100	100	100
(HA2 421–435)			
**LVLLENERTLDYHDS**	100	98	100
(HA2 426–440)			
**IYQILAIYSTVASSL**	93	100	100
(HA2 510–524)			
**YQILAIYSTVASSLV**	100	100	100
(HA2 511–525)			
**STVASSLVLLVSLGA**	93	100	98
(HA2 518–532)			
**GAISFWMCSNGSLQC**	100	98	99
(HA2 531–545)			

Bold faced epitopes were used in *in vitro* assays.

**Table 2:** Predicted DRB1 alleles and other DRB haplotypes as potential MHC II molecules to CD4+ HA T cell epitopes

**T- cell epitopes Position on HA protein**	**Predicted DRB1 alleles**		**Predicted alleles of other DR haplotypes (DRB3, 4 and 5)**	
	**Alleles predicted to bind strongly with each epitope based on threshold binding affinity, <50 nM.**	**Alleles predicted to bind weakly with each epitope based on threshold binding affinity, >50 nM to <500 nM.**	**Alleles predicted to bind strongly with each epitope based on threshold binding affinity, <50 nM.**	**Alleles predicted to bind weakly with each epitope based on threshold binding affinity, >50 nM to <500 nM.**
**HA1 19-33**	DRB1*0701,DRB1*1201, DRB1*1301	DRB1*0101,DRB1*0801,	DRB5*0202	DRB3*0201,DRB3*0301
		DRB1*1401		
**HA1 99-113**	-	DRB1*0101,DRB1*0701,	DRB5*0202	DRB3*0201,DRB4*0101
		DRB1* 1001		DRB5*0101
**HA1 104-119**	-	DRB1*0701,DRB1*1201	-	DRB5*0101,DRB5*0202
**HA1 315-329**	DRB1*0101,DRB1*0801,	DRB1*0401,DRB1*0404,	DRB3*0201,DRB5*0202	DRB3*0301,DRB4*0101
	DRB1*1201	DRB1*0701,DRB1*0901,		DRB5*0101
		DRB1*1001,DRB1*1101,		
		DRB1*1301,DRB1*1401,		
		DRB1*1501		
HA1 317-331	DRB1*0801,DRB1*1201	DRB1*0101,DRB1*0401,	DRB5*0202	DRB3*0201,DRB3*0301
		DRB1*0701,DRB1*1101,		DRB4*0101,DRB5*0101
		DRB1*1301,DRB1*1401,		
		DRB1*1501		
**HA1 319-333**	DRB1*1201	DRB1*0701,DRB1*0801,	DRB5*0202	DRB3*0201,DRB4*0101
		DRB1*1301,DRB1*1501		
HA1 325-339	DRB1*0101	DRB1*0701,DRB1*1201,	-	DRB5*0101,DRB5*0202
		DRB1*1501		
**HA2 377-391**	-	DRB1*0801,DRB1*1201,	-	DRB5*0202
		DRB1*1301		
HA2 382-396	-	DRB1*0101,DRB1*0401,	DRB5*0202	DRB3*0201
		DRB1*0801,DRB1*1201		
**HA2 413-427**	-	DRB1*0101,DRB1*0401,	-	DRB3*0301,DRB5*0202
		DRB1*0404,DRB1*0701,		
		DRB1*1001,DRB1*1201,		
		DRB1*1301,DRB1*1501		
HA2 419-433	-	DRB1*0101	-	DRB3*0301,DRB5*0202
HA2 420-434	-	DRB1*0101,DRB1*0401,	-	DRB3*0201,DRB4*0101
		DRB1*0404,DRB1*1201		DRB5*0202
**HA2 421-435**	DRB1*0101	DRB1*0401,DRB1*0404,	DRB5*0202	DRB3*0201,DRB3*0301
		DRB1*1001,DRB1*1101,		DRB4*0101
		DRB1*1201,DRB1*1301,		
		DRB1*1501		
**HA2 426-440**	DRB1*0101	DRB1*0301,DRB1*0401,	-	DRB3*0201,DRB3*0301
		DRB1*1001,DRB1*1201,		DRB4*0101,DRB5*0202
		DRB1*1301		
**HA2 510-524**	DRB1*0101,DRB1*0404,	DRB1*0401,DRB1*0901,	DRB5*0202	DRB3*0201,DRB3*0301
	DRB1*0701,DRB1*0801,	DRB1*1101,DRB1*1301		DRB4*0101,DRB5*0101
	DRB1*1001,DRB1*1201			
**HA2 511-525**	DRB1*0101,DRB1*0401,	DRB1*1101	DRB3*0201,DRB3*0301	DRB4*0101,DRB5*0101
	DRB1*0404,DRB1*0701,		DRB5*0202	
	DRB1*0801,DRB1*0901,			
	DRB1*1001			
**HA2 518-532**	-	DRB1*0101,DRB1*0701,	-	DRB3*0201,DRB3*0301
		DRB1*1001,DRB1*1201		DRB5*0202
**HA2 531-545**	DRB1*0101	DRB1*0401,DRB1*0404,	-	DRB3*0301,DRB5*0101
		DRB1*0701,DRB1*0901,		DRB5*0202
		DRB1*1001,DRB1*1201,		
		DRB1*1501		

NetMHCIIPan tool was used to predict binding affinity of each epitope with DRB1 alleles and other alleles of DR haplotypes (binding affinity: <50 nM (strong) and >50 nM to <500 nM (weak)). Bold faced epitopes were used in *in vitro* assays.

### Recruitment of human donors and blood collection

This study was performed at York University, Toronto, Canada with appropriate approvals from the Institutional Review Board and Institutional Biosafety Committee during January 2010 to December 2010. Fourteen healthy human donors with an age range of 22 to 66 years were recruited randomly and all donors were provided with informed consent forms. In order to gather information on previous influenza exposure history (immunizations and/or influenza-like illness) of each donor, a questionnaire was provided. Approximately 20 ml of venous blood was drawn from each donor. Also, approximately 20 ul of capillary blood was obtained for the QIAcard FTA spots (QIAGEN Inc. Ontario, Canada) from each donor and were stored in pouches at room temperature after drying.

### Peripheral blood mononuclear cells (PBMC) and plasma isolation

Immediately after blood draw from each donor, PBMCs were isolated and purified using Ficoll-Hypaque gradient centrifugation followed by cryopreservation in liquid nitrogen using 40% Roswell Park Memorial Institute (RPMI) medium, endotoxin free 50% fetal bovine serum and 10% dimethyl sulfoxide (DMSO). Plasma was stored in appropriate tubes (BD Biosciences, Franklin Lakes, and NJ) at -80°C. More descriptive methodology is provided in Additional file 1.

### IFNγ-ELISPOT assay

Enzyme-linked immunospot assay kits (human IFN-γ ELISPOT^PRO^ kits from Mabtech, Nacka Strand, Sweden) were used to determine the frequency of epitope-specific IFN-γ secreting T cells in PBMCs from donors. ELISPOT assays were set up in duplicates. Each SFU corresponds to one IFNγ-secreting T cell. Data are presented as the mean SFU per 2 × 10^5^ PBMCs. The negative control was PBMCs in medium without epitope stimulation and was used to assess the spontaneous secretion of IFNγ. The positive control was polyclonal activator anti-CD3 monoclonal antibody, which helps in determining T cell numbers and viability and functionality of the immunoassay. To increase the reliability of T cellular responses, each synthetic epitope was incubated overnight in ELISPOT plates (without the cells) to detect any non-specific reactions [13]. More descriptive methodology is provided in Additional file 2.

### IgG Enzyme-linked immunosorbent assay (IgG ELISA)

Indirect enzyme-linked immunosorbent assay was developed to quantify specific IgG antibodies to the seasonal H1N1 and the A (H1N1)pdm09 in the donor plasma samples. Hemagglutinin of seasonal H1N1 (2008–2009 vaccine strain, A/Brisbane/59/2007) and A (H1N1)pdm09 (A/California/4/2009) were used as antigens in the ELISA assay (Sino Biological Inc., China). Absorbance at 405 nm was measured immediately with an ELISA microplate reader. Absorbance values correspond to the binding strength of IgG to influenza HA antigens. Assay performance was validated with the human IgG standard in the well, considered as positive control. The negative control was PBS without antigen in the well. The IgG concentration of the samples was determined using a standard curve based on the human IgG standard. Absorbance values two times higher than the absorbance of the negative control were considered to be positive. More descriptive methodology is provided in Additional file 3.

### HLA-DRB1 typing

Low resolution PCR kits with sequence specific primers for HLA-DR with Taq (Olerup, Stockholm, Sweden) were used to determine the DRB1 alleles of each donor. Olerup specific interpretation and specificity tables were used for this purpose.

### Statistical analysis

Unpaired t-test (two-tailed p-value) was applied to compare the response among groups using Graphpad Prism version 5.0.

## Results

### Donors’ flu exposure history and their HLA types

The donors were divided into two cohorts based on their age: D1 to D8 (age range 22 – 34 years, median 24) and D9 to D14 (age range 51 – 66 years, median 56.5). Table 3 presents the results of IgG ELISA (used to identify the donors’ flu exposure) and DRB1 alleles of donors based on HLA-typing. IgG ELISA results revealed that all 14 donors showed the presence of IgG antibodies specific to seasonal H1N1-HA (A/Brisbane/59/2007). However, only five donors had A (H1N1)pdm09 (A/California/04/2009) specific IgG antibodies, and these had all been immunized with the 2009 flu vaccine. Antibody responses to seasonal H1N1 virus antigen were expected as seasonal H1N1 has been a co-circulating seasonal strain for the past 33 years and was widely disseminated in the population through natural infections and annual seasonal vaccinations. IgG ELISA data on donor's flu exposure history corroborates previous seroprevalence studies showing the absence of detectable antibodies [2].

**Table 3:** Information on donors

**Donor ID (age/ gender)**	**Flu vaccination**		**Flu-like exposure**	**IgG-ELISA**		**MHC II (DRB) typing**	
	**Seasonal H1N1**	**A (H1N1)pdm09**		**Seasonal H1N1**	**A (H1N1)pdm09**	**Alleles of DRB1**	**Alleles of DR haplotypes (DRB3, 4 and 5)**
D1 (22/M)	no	no	not sure	yes	no	DRB1*13,	DRB3*52
						DRB1*14	
D2 (22/M)	no	no	yes	yes	no	DRB1*04,	DRB4*53,
						DRB1*15	DRB5*51
D3 (23/F)	no	no	Infection (11/2006)	yes	no	DRB1*04,	DRB4*53,
						DRB1*15	DRB5*51
D4 (25/M)	no	no	yes	yes	no	DRB1*07	DRB3*52
D5 (23/M)	no	no	yes	yes	no	DRB1*03	DRB3*52
D6 (27/M)	no	no	yes	yes	no	DRB1*04,	DRB3*52,
						DRB1*17	DRB4*53
D7 (30/F)	no	yes	Infection (06/2009)	yes	yes	DRB1*08,	DRB5*51
						DRB1*15	
D8 (34/M)	no	yes	No	yes	yes	DRB1*07	DRB4*53
D9 (51/F)	no	no	Infection (07/2010)	yes	no	DRB1*07,	DRB3*52,
						DRB1*17	DRB4*53
D10 (53/M)	no	no	no	yes	no	DRB1*07,	DRB4*53
						DRB1*13	
D11(53/F)	no	no	yes	yes	no	DRB1*07,	DRB3*52,
						DRB1*17	DRB4*53
D12 (60/F)	yes	yes	yes	yes	yes	DRB1*03	DRB4*52
D13 (66/F)	yes	yes	yes	yes	yes	DRB1*15	DRB4*53,
							DRB5*51
D14 (66/M)	yes	yes	yes	yes	yes	DRB1*04	DRB3*52,
							DRB4*53

### CD4+ T-cell memory responses to conserved epitopes

To identify the prevalence of any pre-existing CD4+ T cell immunity we used the direct IFN-γ ELISPOT assay. When PBMCs (2 × 10^5^) of each healthy donor were incubated over night with each of the synthetic epitopes in IFN-γ ELISPOT plates, we observed epitope-specific CD4+ T cell responses. We considered that the CD4+ T cell response to each of the conserved epitopes is a recall CD4+ T cell response. IFN-γ secretions from overnight IFN-γ-assays ensures that the responses are from *in vivo* sensitized CD4+ T cells to these epitopes via natural infection and/or vaccinations, i.e. memory CD4+ T cells rather than by naïve CD4+ T cells expanded *in vitro* in response to the *in vitro* presented epitopes.

Thirteen out of 14 healthy donors generated IFN-γ CD4+ T cell memory responses to the predicted CD4+ T cell epitopes (Table 4). Of these donors, donor D10 responded to 12/14 epitopes. Donors D1 and D13 responded to only one epitope (518–532). D8 showed very low response to two epitopes 510–524 and 511–525 just above the cut-off (10 and 11 SFU respectively). The IFN-γ producing T cell variation ranged from 10 ± 1.9 SFU to 89 ± 2.3 SFU per 2 × 10^5^ PBMCs among all donors that responded. The CD4+ T cellular memory responses of highest magnitude were observed for HA2 epitopes, 511–525, 518–532 and 531–545 with mean SFU across all responding donors as: 24.1, 31.4 and 27.1 respectively. Details on mean number of responding T cells for all epitopes are given in Table 5. Only four donors out of fourteen donors (D5, D10, D11, and D14) responded to HA1 conserved epitopes and the responses were of a low magnitude (11 ± .22 SFU to 19 ± 2.3 SFU). Older age-group showed higher magnitude of T-cell responses to epitopes when compared to younger group.

**Table 4:** T cell response of donors to predicted and conserved T cell epitopes encompassing entire HA protein

**Donor ID (age/ gender)**	**HLA-DRB1, 3, 4, and 5 alleles**	**CD4+ epitopes on HA protein**												
		**HA1 (1–329 a.a)**					**HA2 (330–561 a.a)**							
		**19-33**	**99-113**	**104-119**	**315-329**	**319-333**	**377-391**	**413-427**	**421-435**	**426-440**	**510-524**	**511-525**	**518-532**	**531-545**
D1 (22/M)	DRB1*13												X	
	DRB1*14													
	DRB3*52													
D2 (22/M)	DRB1*04,												X	X
	DRB1*15													
	DRB4*53,													
	DRB5*51													
D3 (23/F)	DRB1*04,													
	DRB1*15													
	DRB4*53,													
	DRB5*51													
D4 (25/M)	DRB1*07												X	X
	DRB3*52													
D5 (23/M)	DRB1*03					X		X					X	X
	DRB3*52													
D6 (27/M)	DRB1*04,												X	X
	DRB1*17													
	DRB3*52,													
	DRB4*53													
D7 (30/F)	DRB1*08,						X				X	X	X	X
	DRB1*15													
	DRB5*51													
D8 (34/M)	DRB1*07										X	X		
	DRB4*53													
D9 (51/F)	DRB1*07,						X		X	X			X	
	DRB1*17													
	DRB3*52,													
	DRB4*53													
D10 (53/M)	DRB1*07,			X	X	X	X	X	X	X	X	X	X	X
	DRB1*13													
	DRB4*53													
D11 (53/F)	DRB1*07,				X	X			X	X	X	X	X	X
	DRB1*17													
	DRB3*52,													
	DRB4*53													
D12 (60/F)	DRB1*03										X	X	X	X
	DRB4*52													
D13 (66/F)	DRB1*15												X	
	DRB4*53,													
	DRB5*51													
D14 (66/M)	DRB1*04	X		X	X	X	X					X	X	X
	DRB3*52,													
	DRB4*53													

X: denotes positive IFN-γ secretions.

**Table 5:** Mean number of responding T cells across donors responding to the given HA epitope

**HA epitopes**	Total responding T cells* (SFU)	**No of donors responded**	**Mean number of responding T cells (SFU)**
19-33	11.5	1	11.5
99-113	0.0	0	0.0
104-119	25.5	2	12.8
315-329	45.5	3	15.2
319-333	38.0	3	12.7
377-391	66.0	4	16.5
413-427	25.5	2	12.8
421-435	58.0	3	19.3
426-440	33.0	3	11.0
510-524	73.5	5	14.7
511-525	144.5	6	24.1
518-532	376.5	12	31.4
531-545	243.5	9	27.1

* Only positive responding T cells (≥ 10) were considered.

### Immunodominance of CD4+ T cell HA epitopes

Table 4 tabulates the percentage of donors who elicited CD4+ T cellular memory responses to highly conserved HA epitopes. There are two criteria set for “immunodominant epitopes” i.e., high frequency of response in population (>50%) and a high intensity of response as compared to all the epitopes tested [14,15]. In our study, two of the 14 epitopes, 518–532 and 531–545 were identified as “immunodominant” as they elicited CD4+ T cell response from 85% (12/14) and 71% (10/14) of donors respectively. In addition, these two epitopes (518–532 and 531–545) elicited the highest magnitude of CD4+ responses among all tested epitopes; the number of IFN-γ producing CD4+ T cells being 89 ± 2.3 and 80 ± 3.3 SFU per 2×10^5^ PBMCs respectively. Epitopes, 511–524 (43%), 510–524 (36%), 377–391 (29%) and 319–333 (29%) met the criteria to be sub-dominant epitopes based on the frequency of donors with CD4+ T cell responses i.e. >25% to <50%.

### Influence of MHC class II alleles on CD4+ T cellular response to conserved HA epitopes

The strongest CD4+ T cellular memory responses were seen from donor 11 (D11) who has the DRB1*07 and DRB1*17 alleles (Table 4). D11 showed CD4+ T cell response to epitopes 315–329, 319–333, 421–435, 426–440, 510–524, 511–525, 518–532 and 531–545. D4, is identical to D11 with regards to DRB1*07. The strongest responses of D4 were to epitopes 518–532 and 531–545 but no response was seen to the other epitopes to which D11 responded. D8 who also has the DRB1*07 allele responded to epitopes 510–524 and 511–525 (albeit weakly).

**Table 6:** Information on percentage of donors, and their T cell response and DRB MHC type of donors responding to predicted and conserved T cell epitopes

**HA epitope and position**	**% donors responding to each epitope (responded donors/total donor**	**Total responding T cells**	**Mean number of responding T cells**	**DRB1 alleles of donors**	**Alleles of other DR haplotypes of donors**
STVASSLVLLVSLGA	85 (12/14)	376.5	31.4	DRB1*03, DRB1*04,	DRB3*52,
(518–532)				DRB1*07, DRB1*08,	DRB4*53,
				DRB1*13, DRB1*14,	DRB5*51
				DRB1*15, DRB1*17	
GAISFWMCSNGSLQC	71 (9/14)	243.5	27.1	DRB1*03, DRB1*04,	DRB3*52,
(531–545)				DRB1*07, DRB1*08,	DRB4*53,
				DRB1*13, DRB1*15,	DRB5*51
				DRB1*17	
YQILAIYSTVASSLV	43 (6/14)	144.5	24.1	DRB1*04, DRB1*07,	DRB3*52,
(511–525)				DRB1*08, DRB1*13,	DRB4*53,
				DRB1*15, DRB1*17	DRB5*51
IYQILAIYSTVASSL	36 (5/14)	73.5	14.7	DRB1*07, DRB1*08,	DRB3*52,
(510–524)				DRB1*13, DRB1*15,	DRB4*53,
				DRB1*17	DRB5*51
NKVNSVIEKMNTQFT	29 (4/14)	66.0	16.5	DRB1*04, DRB1*07,	DRB3*52,
(377–391)				DRB1*08, DRB1*13,	DRB4*53,
				DRB1*15, DRB1*17	DRB5*51
RNIPSIQSRGLFGAI	29 (4/14)	38.0	12.7	DRB1*03, DRB1*04,	DRB3*52,
(319–333)				DRB1*07, DRB1*13,	DRB4*53
				DRB1*17	
LVLLENERTLDYHDS	21 (3/14)	33.0	11.0	DRB1*07,DRB1*13,	DRB3*52,
(426–440)				DRB1*17	DRB4*53
YNAELLVLLENERTL	21 (3/14)	58.0	19.3	DRB1*07,DRB1*13,	DRB3*52,
(421–435)				DRB1*17	DRB4*53
ATGLRNIPSIQSRGL	21 (3/14)	45.5	15.2	DRB1*04, DRB1*07,	DRB3*52,
(315–329)				DRB1*13, DRB1*17	DRB4*53
DGFLDIWTYNAELLV	14 (2/14)	25.5	12.8	DRB1*03, DRB1*07,	DRB3*52,
(413–427)				DRB1*13,	DRB4*53
QLSSVSSFERFEIFP	14 (2/14)	25.5	12.8	DRB1*04, DRB1*07,	DRB3*52,
(104–119)				DRB1*13	DRB4*53
VLEKNVTVTHSVNLL	7 (1/14)	11.5	11.5	DRB1*04	DRB3*52,
(19–33)					DRB4*53
EELREQLSSVSSFER	No response	-	-	-	-
(99–113)					

On the other hand, D10 who also has DRB1*07 but in addition, DRB1*13, showed a positive response to many epitopes in common with D11 along with unique responses to some epitopes (Table 4). Overlap in their CD4+ T cell responses was seen for eight out of 11 epitopes for D10 and D11. Four epitopes were unique to D10 (who expresses DRB1*13 along with DRB1*07).

Next we examined responses from donors expressing the DRB1*04 allele. D14 who expresses the DRB1*04 allele responded to epitopes 19–33, 315–329, 319–333, 377–391, 511–525, 518–532 and 531–545. Donors D2 and D3 both have DRB1*04 and DRB1*15 alleles. However, only D2 responded to epitopes 518–532 and 531–545. D6 responded to epitopes 518–532 and 531–545 and expressed DRB1*04 and DRB1*17 alleles. D7 who expressed DRB1*08, DRB1*15 responded to epitopes of 377–391, 510–524, 511–525, 518–532, and 531–545. D12 who expressed DRB1*03 allele responded to epitopes 510–524, 511–525, 518–532, and 531–545. In contrast to other donors, D13 was responsive only to the epitope 518–532. We recognized that two HA2 epitopes 518–532 and 531–545 exhibited high promiscuity since they generated response in donors irrespective of their MHC backgrounds. The presenting MHC II alleles listed in the descending order of their promiscuity with epitopes, are DRB1*07, DRB1*04, DRB1*15 > DRB1*03, DRB1*17 > DRB1*13, DRB1*14, and DRB1*08. To summarize, there are instances with donors with same MHC allele background however responding to different epitopes and donors with different MHC alleles present same epitopes (Table 6).

## Discussion

Various immuno-informatics studies revealed high degree of T cell-specific epitope conservancy between seasonal H1N1 and A(H1N1)pdm09 [5,6,12]. *Invitro* and *invivo* T cellular functional studies assumed that common and shared epiope-specific T-cell responses are involved in the attenuation of disease severity of A (H1N1)pdm09 infection [6,14,15]. In the current study, we have conducted T cellular functional assays and HLA-typing (MHC II-DRB1) experiments to identify the immunodominant epitopes of conserved HA CD4+ T cell epitopes between seasonal H1N1 and A (H1N1)pdm09.

The over-night T cell functional assays revealed that the CD4+ T cellular memory responses could readily be generated to highly conserved HA epitopes from majority of the donors (93%, 13/14). However, we found variability in reactivity to HA CD4+ T cell epitopes among donors, which could be correlated to factors such as age, previous exposure history to similar flu viral strains, genetics i.e. MHC class II allele promiscuity, and the binding affinity between MHC and its epitope [14,15]. Older donors showed significantly higher magnitude and diversified CD4+ T cellular responses when compared to young (mean SFU across responding donors 136.5 vs. 41.6, unpaired t-test two-tailed p-value = 0.0060). It is very likely that older adults have larger and more varied repertoire of CD4+ T memory cells due to repeated seasonal H1N1 exposures. We also observed the highest immunogenic (CD4+) responses from donors who expressed DRB1*07, DRB1*04, DRB1*15 alleles. Of particularly DRB1*07 was shown to present and elicit CD4+ T cell responses for 86% (12/14) of epitopes tested.

From our *in vitro* analysis, we have identified two immunodominant epitopes (518–532 and 531–545) from the stalk region of HA protein. Epitope, 531–545, GAISFWMCSNGSLQC is a unique CD4+ T cell epitope that has not identified previously as being immunogenic. The immunodominance of these two epitopes is likely due to their localization in a highly conserved stalk (HA2) region of HA, and their high promiscuity with DRB1 alleles. Extensive promiscuity of epitopes to different MHC alleles increases their likelihood of being presented by majority of population and more likely to be recognized by a broad CD4+ T cell repertoire. We also observed that same alleles often present very different epitopes. This represents that some MHC alleles can exhibit less restrictive peptide binding and this type of phenomena have been observed before [16]. Despite the presence of a similar MHC background, the availability of T cell repertoire that recognizes a particular epitopes determines the response to a given epitope.

Interestingly, immunodominance does not correlate with the binding affinities of peptide- DRB1*07. Similar poor correlation results were also documented from other studies on CD4+ T cell responses to epitopes of influenza A viruses [17] and other antigens [18-20]. This poor correlation can possibly be influenced by the antigen abundance and the T cell repertoire. With relevance to available T cell repertoire on immunodominance, previous studies have clearly shown that epitope hierarchy from subdominant to immunodominant could be altered by pre-immunization with specific epitopes and thereby increase the frequency of T cells [21,22]. As a result of repeated exposures to similar Influenza A viruses, T cell repertoire can be considerably generated to the highly conserved regions as shown in our study. A recent study by Wilkinson et al. [23], has shown evidence of pre-existing influenza-specific CD4+ T cell response to internal proteins, with cytotoxic activity in reducing viral shedding and mitigating illness in human volunteers.

The current study provides compelling evidence of epitope specific CD4+ T cellular memory albeit with some experimental limitations. Based on extensive studies on optimal size of epitope (15 a.a. length) for CD4+ T cells we have assumed that the observed T cellular memory responses are from CD4+ T cells [24]. Additional evidence for our assumption comes from experimental reports showing CD4+ T cellular responses to conserved regions of influenza A virus proteins with memory markers [6,14]. However, we cannot rule out the possibility of low level responses from CD8+ T cells to these conserved epitopes. We also acknowledge that serology by ELISA is not an ideal way to determine the exposure to flu; the ideal way would have been to have laboratory confirmed influenza infection. Further the immunogenic conserved HA epitopes identified in the current study have to be validated on a larger scale to characterize and reconfirm the epitope immunodominance.

## Conclusion

Influenza A viruses belongs to a group of the best studied viruses, yet they pose a challenge to the human population in the form of epidemics and pandemics. One of the drawbacks of current available seasonal influenza trivalent vaccines is that they can elicit potent neutralizing antibody responses against vaccine strains and closely related antigenic viral strains but not against antibody-escape variants like novel or pandemic strains. This limited efficacy of vaccines necessitates annual reformulation of seasonal vaccines. The greatest threat, however, is during pandemics when most of the population is naïve and thus the pandemic could lead to considerable morbidity and mortality. As shown in our study, there could be considerable role of pre-existing T cellular immunity in modulating the disease severity of pandemic strains even in the absence of pre-existing neutralizing antibodies. Our study also supports the need to develop vaccines with conserved CD4+ T cell epitopes to increase the efficacy of vaccines. One of the challenges of CD4+ T cell epitope based vaccines is MHC class II restriction and polymorphism in humans. However, our study has shown that by large scale bioinformatics analysis it is possible to identify CD4+ T cell epitopes that promiscuously bind to several MHC class II alleles thus have potential for maximal population coverage.

## Competing interests

Dr. Gubbay has received research grants from GlaxoSmithKline Inc. and Hoffman-La Roche Ltd to study antiviral resistance in influenza, and from Pfizer Inc. to conduct microbiological surveillance of Streptococcus pneumoniae. All other co-authors do not have any conflict of interest.

## Authors’ contributions

Conceived and designed the experiments: VRD, BD, GEW; Performed the experiments: VRD, BD; Analyzed the data: VRD, BD; Contributed reagents/materials/analysis tools: VRD, BD, RJ, JBG, JW, GEW; Wrote the paper: VRD, BD, VJ, JBG, GEW. All authors read and approved the final manuscript.

## Authors’ information

Venkata R. Duvvuri and Bhargavi Duvvuri joint first authorship.

## Pre-publication history

The pre-publication history for this paper can be accessed here:

http://www.biomedcentral.com/1471-2334/13/204/prepub

## Supplementary Material

Additional file 1 Detailed methodology of Peripheral blood mononuclear cells (PBMC) and plasma isolation. Click here for file

Additional file 2 Detailed methodology of IFN-γ ELISPOT^PRO^ assay. Click here for file

Additional file 3 Determination of flu exposures with Human IgG Enzyme-linked immunosorbent assay. Click here for file
