# Supplementary material for: T cell memory to evolutionarily conserved and shared hemagglutinin epitopes of H1N1 viruses: a pilot scale study
Source: BMC Infect Dis. 2013 May 4;13:204. doi: 10.1186/1471-2334-13-204 (PMC3649888; doi:10.1186/1471-2334-13-204)
Supplement: Additional file 1 — Detailed methodology of Peripheral blood mononuclear cells (PBMC) and plasma isolation. [file 1471-2334-13-204-S1.docx]

**Additional Files 1.**

**Detailed methodology of Peripheral blood mononuclear cells (PBMC) and plasma isolation:**

Blood was diluted with an equal amount of 1X Mg^++^ and Ca^++^ free phosphate buffered saline (PBS) room temperature (RT) and was mixed gently. 15 ml of RT Ficoll was added to a sterile 50 ml conical tube. Ficoll was gently overlaid with 30 ml of diluted blood using a sterile serological pipette. Mixing of two phases was kept to the minimum followed by centrifugation at 591 relative centrifugal force (RCF) for 30 minutes at RT with brake off to ensure that deceleration does not disrupt the density gradient. Using a Pasteur pipette PBMC (cloudy layer) from the diluted plasma/Ficoll interface was collected and cells were placed into a sterile 50 ml conical tube. Interface from a maximum of two 50 ml tubes were combined into one wash tube. While collecting cells, care was taken to aspirate as little Ficoll as possible since lower cell number will pellet if the proportion of Ficoll is too high in the wash tube. 1X PBS was added to the PBMC layer to bring up the volume to 45 ml followed by mixing gently up and down. Mixture was centrifuged at 330 RCF for 10 minutes at RT with brake ON. Following centrifugation supernatant was removed and discarded without touching the pellet. Pellet was loosened by tapping the tube with finger and 5ml of sterile 1X PBS+1% fetal bovine serum (FBS) (Freezing media A) was added and carefully mixed by gently pipetting up and down. All individual suspensions were pooled into a single sterile 50 ml tube. Cells were mixed and 20 µl of the cell suspension was taken out for cell counting. Total number of cells was counted. Cell suspension was centrifuged at 330 RCF for 10 minutes at RT with brake ON. Supernatant was discarded and to the pellet appropriate amount of Freezing media A was added to adjust the cell concentration to 20 x 10^6^/ml. Cells were re-suspended by tapping the tube with finger until no pellet was visible. Slowly, drop by drop an equal volume of Freezing media B (20% Dimethyl sulfoxide (DMSO) in FBS) was added to the side of tube containing Freezing media A containing the PBMCs. To mix the cells, cell suspension was gently mixed by pipetting up and down and avoiding bubbles. The final concentration was maintained to be 10 x 10^6^cells/ml. Once mixing was complete PBMC suspension was aliquoted into the pre-labled crovials, 0.5-1ml in 1.8 ml vial (~5-10 million cells per vial and no more than 10 million cells per vial). Cells were gently pipetted to minimize the sheer forces. Cryovials were kept in -70^o^C freezer for at least 12 hours and followed by final transfer to liquid nitrogen for indefinite storage.
