# Supplementary material for: T cell memory to evolutionarily conserved and shared hemagglutinin epitopes of H1N1 viruses: a pilot scale study
Source: BMC Infect Dis. 2013 May 4;13:204. doi: 10.1186/1471-2334-13-204 (PMC3649888; doi:10.1186/1471-2334-13-204)
Supplement: Additional file 2 — Detailed methodology of IFN-γ ELISPOTPRO assay. [file 1471-2334-13-204-S2.docx]

**Additional File 2.**

**Detailed methodology of IFN-γ ELISPOT^PRO^** **assay:**

Enzyme-linked immunospot assay kits (human IFN-**γ** ELISPOT^PRO^ kits, Mabtech, (Nacka Strand, Sweden)) were used to determine the frequency of epitope-specific IFN-γ secreting T cells. PBMCs (2 x 10^5^ per well) were pulsed with peptides (10 µM) O/N in polyvinlylidene difluoride plates pre-coated with anti-human IFN-γ mAb at 37^0^C in 5% CO2. After O/N incubation, wells were washed with filtered PBS to remove the non- adherent cells. Following washing, each well was treated with 100 µl of one step-detection reagent (alkaline phosphatase (ALP)-conjugated detection IFNγ antibody) 1:200 in filtered PBS containing 0.5% FBS, for 2 hrs at 37^0^C. Plates were washed five more times with filtered PBS before chromogenic development with 100 µl/well “ready to use” 5-Bromo-4-chloro-3-indolyl phosphate (BCIP) / nitro-blue tetrazolium chloride (NBT). BCIP/NBT-added plates were incubated for 15min at RT during which time the blue spots developed. Each spot-forming unit (SFU) corresponds to one IFNγ-secreting T cell. The numbers of antigen specific T cells are presented as SFU per total number of PBMCs added. The negative control was PBMCs in medium without peptide stimulation and was used to assess the spontaneous secretion of IFNγ. The positive control was polyclonal activator anti-CD3 mab (>500 SFU/2 x 10^5^ PBMCs are expected) and was used in determining T cell numbers, viability and functionality of the immunoassay. Each peptide was incubated O/N in ELISPOT plates without PBMCs to detect non-specific reactions. A well was considered positive when it contained at least 10 SFU and twice as many SFU as the negative control wells. Each data point is the mean of duplicate wells.
