# Supplementary material for: T cell memory to evolutionarily conserved and shared hemagglutinin epitopes of H1N1 viruses: a pilot scale study
Source: BMC Infect Dis. 2013 May 4;13:204. doi: 10.1186/1471-2334-13-204 (PMC3649888; doi:10.1186/1471-2334-13-204)
Supplement: Additional file 3 — Determination of flu exposures with Human IgG Enzyme-linked immunosorbent assay. [file 1471-2334-13-204-S3.docx]

**Additional File 3.**

**Determination of flu exposures with Human IgG Enzyme-linked immunosorbent assay:**

Indirect ELISA (from MABTECH) was used to quantify specific IgG antibodies to the seasonal H1N1 and the 2009 pandemic swine H1N1 in the donor plasma samples. Hemagglutinin antigens of seasonal H1N1 (A/Brisbane/59/2007, Catalogue Number: 11052-V08H) and 2009 pandemic H1N1 (A/California/4/2009, Catalogue Number: 11055-V08H) were procured from the Sino Biological Inc, China. ELISA plates were coated overnight at 40C with 100ul per well of 5μg/ml of the appropriate influenza virus antigens dissolved in phosphate buffered saline, pH 7.4 (PBS). After overnight incubation, plates were washed twice with PBS. Plates were blocked by adding 200μl per well of *PBS with 0.1% of fetal bovine serum (FBS) (*incubation buffer) and incubated for 1 hour at room temperature (RT). The plates were washed and incubated with 100μl per well of plasma (1:1 diluted in incubation buffer) for 2hrs at RT. After washing, plates were incubated for 1 hr at RT with 100μl per well of IgG isotype-specific mouse anti-human conjugated to alkaline phosphatase. After five washes, the plates were incubated with 100μl per well of substrate solution: p-nitrophenyl-phosphate (pNPP) for 30 minutes in the dark. Absorbance at 405nm was measured immediately with an ELISA microplate reader. Absorbance values correspond to the binding strength of IgG to influenza HA antigens. This reaction can be stopped by adding equal volume of 0.75 M NaOH if necessary. The positive control was a human IgG standard in the well to validate the assay performance. The negative control was PBS without antigen in the well. The control wells were treated the same as the experimental wells. IgG content (concentration) of samples was determined from the standard curve based on the human IgG standard. Absorbance values two times higher than the absorbance of the negative control were considered to be positive.
